# Contributions of common genetic variants to specific languages and to when a language is learned

**DOI:** 10.1038/s41598-021-04163-1

**Published:** 2022-01-12

**Authors:** Patrick C. M. Wong, Xin Kang, Hon-Cheong So, Kwong Wai Choy

**Affiliations:** 1 Department of Linguistics and Modern Languages, The Chinese University of Hong Kong, Shatin, Hong Kong SAR China; 2 Brain and Mind Institute, The Chinese University of Hong Kong, Shatin, Hong Kong SAR China; 3 Department of Otorhinolaryngology, Head and Neck Surgery, The Chinese University of Hong Kong, Shatin, Hong Kong SAR China; 4 Research Centre for Language, Cognition and Language Application, Chongqing University, Chongqing, China; 5 School of Foreign Languages and Cultures, Chongqing University, Chongqing, China; 6 School of Biomedical Sciences, The Chinese University of Hong Kong, Shatin, Hong Kong SAR China; 7 Department of Obsterics and Gynecology, The Chinese University of Hong Kong, Shatin, Hong Kong SAR China

**Keywords:** Psychology, Human behaviour

## Abstract

Research over the past two decades has identified a group of common genetic variants explaining a portion of variance in native language ability. The present study investigates whether the same group of genetic variants are associated with different languages and languages learned at different times in life. We recruited 940 young adults who spoke from childhood Chinese and English as their first (native) (L1) and second (L2) language, respectively, who were learners of a new, third (L3) language. For the variants examined, we found a general decrease of contribution of genes to language functions from native to foreign (L2 and L3) languages, with variance in foreign languages explained largely by non-genetic factors such as musical training and motivation. Furthermore, genetic variants that were found to contribute to traits specific to Chinese and English respectively exerted the strongest effects on L1 and L2. These results seem to speak against the hypothesis of a language- and time-universal genetic core of linguistic functions. Instead, they provide preliminary evidence that genetic contribution to language may depend at least partly on the intricate language-specific features. Future research including a larger sample size, more languages and more genetic variants is required to further explore these hypotheses.

## Introduction

Even before the publication of the first studies on the molecular genetics of a speech disorder^1, 2^, researchers have hypothesized that developmental speech and language disorders were inherited (see Ludlow and Cooper^3^ for an early review). In the two decades since the first studies concerning *FOXP2* and apraxia of speech were made^1, 2^, a series of studies (e.g., see Newbury and Monaco^4^ for a review) have identified new genes that explained a small portion of variation in spoken and written language functions and disorders^5, 6^. These latter studies often focused on common genetic variants and their associations with language-related traits (e.g., non-word repetition). Though the effect sizes are small, the study of common variants offers an important opportunity to investigate variation of language functions on a continuum. Subtle differences in language functions (e.g., lower proficiency in using a particular set of grammatical forms in language rather than a severe breakdown in communication) are more likely to be associated with primary language impairment and variations in success in acquiring foreign languages. These subtle differences differ from severe forms of speech and language impairment (e.g., childhood of apraxia of speech) that are more likely to be associated with rarer genetic mutations (e.g., Thevenon et al.^7^). The focus of the present study is on common variants and subtle differences in language.

In addition to investigating the molecular pathways that give rise to the neurological functions of genes associated with language functions and disorders^8–10^ and to identifying more new genes, we argue that the genetic studies of language should consider two additional questions concerning variation on a continuum. First, what can the genetics of language inform us about how languages are learned? Second, if an ultimate translational goal of the study of genetics of language is to develop a screening tool for primary language impairment, how can it be used for the more than 7000 languages that are currently spoken and languages that are learned at different times in life?

In both native^11, 12^ and foreign^13, 14^ language learning, a large degree of individual variability in learning success has been observed (see Kidd et al.^15^ for a review). Many factors have been attributed to individual variability, including socioeconomic background for native languages^16^, and memory^17^, music experience^18–20^ and subtle neuroanatomical differences^21^ for foreign languages. At the lower end of individual variability is primary language impairment, which includes Developmental Language Disorder (formerly known as Specific Language Impairment) and dyslexia, which concerns impairment of language in the written modality. The vast majority of the studies were conducted to examine individual variability in native language, and more specifically English and other European languages as a native language.

To obtain a more comprehensive understanding of the genetic basis of language, research must consider not only genetic associations with native language on a continuum of proficiency level, but also foreign languages learned at different time points in life. Such an understanding would give us a clearer idea of whether the genetic effects on language functions are subject to developmental and learning factors. Languages that are learned later in life may require a different set of cognitive resources than languages learned in infancy, which may have been contributory sources of individual variability in L2 attainment^22^. If that is the case, the genes that explain individual differences in native language would not be the same as those in foreign languages. In fact, it may be the case that the genetic effects on native language would be larger than the effects on foreign languages. A better understanding would address long-standing debates in language learning about whether the learning of native and foreign languages is fundamentally different^23^. As far as we are aware, with the exception of Waye et al.^24^, who examined Chinese and English literacy in bilingual children and one gene, no genetic studies of language have yet investigated foreign language learning. Rimfeld et al.^25^ examined the genetic contributions to foreign language learning using a twins sample and did not examine the molecule genetics of such contributions.

A more comprehensive understanding of the genetic basis of language must also investigate languages other than European languages. More than 7000 languages are spoken worldwide^26^. The genes that have been attributed to language could be those that subserve language functions independent of language features (e.g., lexical retrieval, which is required for all languages) or functions that are specific to a linguistic feature (e.g., inflectional morphology, which occurs only in some languages). A real-world implication for understanding the language universal or specific nature of genetic association concerns whether the same genetic diagnosis of language impairment can be made only for a specific language or for any language. In recent years, genetic research has been extended to the examination of non-European languages such as Chinese^27, 28^. However, with the notable exception of the work of Waye et al.^24^, these studies of non-European languages did not examine the genetic associations of two languages within the same population. This makes it difficult to tease apart language and population specific effects, because these two factors often co-vary.

The present study covers young adult participants whose L1 and L2 are Chinese and English, respectively, who were students learning French, German, or Spanish as L3 at college level. The study aims to further our understanding of how common genetic variants are associated with language in three ways. First, while most studies to date on the genetic basis of language have focused on English-speaking individuals (see Devanna et al.^29^ for a review), we asked whether the same genetic variants collectively demonstrate an extended effect on language ability that is measured in early adulthood in speakers of Chinese. To answer this question, we surveyed the literature on the genetic basis of language and identified a group of 28 genetic variants (Table 1). We then simultaneously examined their effects on the participants’ native, first language (L1) as measured by the Chinese subject test of the college entrance examination in Hong Kong. Table 2 summarizes the participant characteristics.

**Table 1:** SNPs of language-related genes hypothesized to be associated with language proficiency that we examined in the present study.

Gene	SNP	Population	Phenotype	Major allele	Minor allele	References	Total N of cases
*ATP2C2*	rs11860694	Majority European (UK)	Non-word repetition in English in SLI individuals	G = 0.76	C = 0.24	^30^	879
*CEP63*	rs7619451	European (Swedish)	Reading comprehension in dyslexic individuals	G = 0.82	T = 0.18	^31^	801
*CMIP*	rs6564903	Majority European (UK)	Non-word repetition in English in SLI individuals	C = 0.80	T = 0.20	^30^	876
*CNTNAP2*	rs2538976	Majority European (UK)	SLI diagnosis in English	T = 0.54	C = 0.46	^5^	881
		European (Australian)	Early communicative behaviour scores in English in SLI individuals			^32^	
*CNTNAP2*	rs2538991	European (UK)	SLI diagnosis in English	C = 0.64	A = 0.36	^5^	880
*COMT*	rs4680	East Asian (Han Chinese)	Immediate memory, visuospatial and language scores in Chinese	G = 0.72	A = 0.28	^33^	871
*DCDC2*	rs1087266	Asian (Uyghur)	Dyslexia diagnosis in Uyghur	A = 0.59	G = 0.41	^34^	739
*DCDC2*	rs2274305	Asian (Uyghur)	Dyslexia diagnosis in Uyghur	C = 0.81	T = 0.19	^34^	791
*DCDC2*	*rs3765502*	Asian (Uyghur)	Dyslexia diagnosis in Uyghur	T = 0.58	C = 0.42	^34^	765
*DCDC2*	rs4599626	Asian (Uyghur)	Dyslexia diagnosis in Uyghur	C = 0.83	A = 0.17	^34^	750
*DCDC2*	rs6456593	Asian (Uyghur)	Dyslexia diagnosis in Uyghur	C = 0.63	G = 0.37	^34^	738
*DCDC2*	rs6940827	East Asian (Han Chinese)	Dyslexia diagnosis in Chinese	G = 0.82	A = 0.18	^35^	766
*DCDC2*	rs807724	Asian (Uyghur)	Dyslexia diagnosis in Uyghur	T = 0.96	C = 0.04	^34^	763
		East Asian (Han Chinese)	Reading fluency, character reading, morphological production and tone deletion in Chinese			^36^	
		Majority European (UK)	Dyslexia diagnosis in English			^37^	
		European (UK)	Single word reading and non-word repetition in English			^6^	
*KIAA0319*	rs9461045	Asian (Uyghur)	Dyslexia diagnosis in Uyghur	T = 0.62	C = 0.38	^27^	807
		Majority European (UK)	Forced word choice test, irregular word coding, and single-word spelling in English in dyslexic individuals			^37^	
		European (UK)	Single word reading and non-word repetition in English			^6^	
		Majority European (UK)	Forced word choice test, irregular word coding, single-word reading and single-word spelling in English			^38^	
*DGKI*	rs889869	European (German)	Dyslexia diagnosis in German	G = 0.84	A = 0.16	^39^	809
*DIP2A*	rs2255526	East Asian (Han Chinese)	Dyslexia diagnosis in Chinese	A = 0.78	G = 0.22	^40^	755
*DYX1C1*	rs3743205	East Asian (Han Chinese)	One minute reading, digit rapid naming, non-word repetition and left–right reversal in Chinese	C = 0.97	T = 0.03	^24, 41^	813
*DYX1C1*	rs57809907	Majority European (UK)	Forced word choice test in English in SLI individuals	C = 0.99	A = 0.01	^37^	811
*DRD2*	rs1800497	European (US)	Artificial grammar learning	G = 0.60	A = 0.41	^42^	816
*DYX1C1*	rs11629841	East Asian (Han Chinese)	Character dictation and orthographic judgment in Chinese	T = 0.96	G = 0.05	^43^	764
*FOXP2*	rs1852469	East Asian (Han Chinese)	Diagnosis of speech sound disorder in Chinese	A = 0.69	T = 0.31	^44^	756
*FOXP2*	rs2396722	East Asian (Han Chinese)	Diagnosis of speech sound disorder in Chinese	T = 0.51	C = 0.49	^44^	739
*FOXP2*	rs6980093	European (Italian)	Semantic fluency and single-word reading in Italian in dyslexic individuals	A = 0.62	G = 0.38	^45^	865
*KIAA0319*	rs3756821	East Asian (Uyghur)	Dyslexia diagnosis in Uyghur	C = 0.77	T = 0.23	^27^	798
		East Asian (Han Chinese)	Dyslexia diagnosis in Chinese			^28^	
		Majority European (US)	General reading skills and text reading in English in SLI individuals			^46^	
*KIAA0319*	rs4504469	East Asian (Han Chinese)	Dyslexia diagnosis in Chinese	C = 0.87	T = 0.13	^47^	804
		Asian (Indians)	Dyslexia diagnosis in Hindi			^48^	
		Majority European (US)	General reading skills in English			^46^	
		Majority European (UK)	Dyslexia diagnosis in English			^49^	
*KIAA0319*	rs807507	East Asian (Han Chinese)	Onset detection test in Chinese in dyslexic individuals	G = 0.80	C = 0.20	^28^	809
*ROBO1*	rs6803202	Majority European (Australian)	Non-word repetition in English	C = 0.59	T = 0.41	^50^	784
*S100B*	rs9722	European (German)	Spelling test in German	G = 0.68	A = 0.32	^51^	797

Major and minor allele frequencies are those in our sample. Examples of relevant studies for each gene are listed under References.

**Table 2:** Demographic information and phenotype scores of participants.

Variables	Mean (SD)	Range	Total N of cases
Gender(F/M)	696/244		940
Musical training (Y/N)	760/172		932
Nonverbal IQ	108.00	85–132	920
Family SES	37.00	1–66	877
L3 age (years old)	19.98	18–25	940
L1	4.94	3–7	640
L2	5.18	3–7	640
L3	− 0.031	− 3.16 to 5.00	857
L3 external motivation	0.016	− 2.61 to 2.89	929
L3 internal motivation	0.001	− 3.41 to 0.65	929
L3 attitude	0.020	− 4.94 to 2.33	926
L3 anxiety	− 0.002	− 3.05 to 1.98	926

L1 and L2 proficiency were represented by the composite grades of the Chinese and English subjects in the HKDSE exam. Gender (F/M) = female/male. Music training (Y/N) = have/have not received at least 1 year of musical training.

Second, we examined whether this same group of common variants, whose effects were studied for native language (cf Vaughn and Hernandez^52^, and Waye et al.^24^ for bilingual speakers), would exert similar effects on a foreign, second language (L2) that was learned since early childhood with a relatively high proficiency. Foreign language proficiency was measured by the English subject test of the same college entrance examination in Hong Kong from the same group of participants. Third, we investigated whether the same genetic variants contribute to the learning of a new, third language (L3) in adulthood. We used the same group of participants, namely students at college-level modern language courses whose L3 ability was measured comprehensively by a composite series of classroom and laboratory tests (see SI for more information).

Our study tests two sets of hypotheses. The first hypothesizes that a group of genetic variants contributes to a set of core language functions that are universal across languages and independent of when learning occurs (whether the learned language is native or foreign). This group of genetic variants would contribute to the learning of L1, L2 and L3. Alternatively, we argue that different languages and languages learned at different times have different genetic underpinnings. As different language features are associated with different brain functions (e.g., the middle frontal gyrus is specific for Chinese reading)^53, 54^, these functions would have different underlying neurogenetic processes. Differences may also be due to the possibility that languages that are learned at different times in life are subject to the influence of different sets of non-genetic factors^55^. For example, the learning of new languages is subject to social factors such as motivation^56^ that may not have the same influence on L1.

## Results

**Figure 1:**
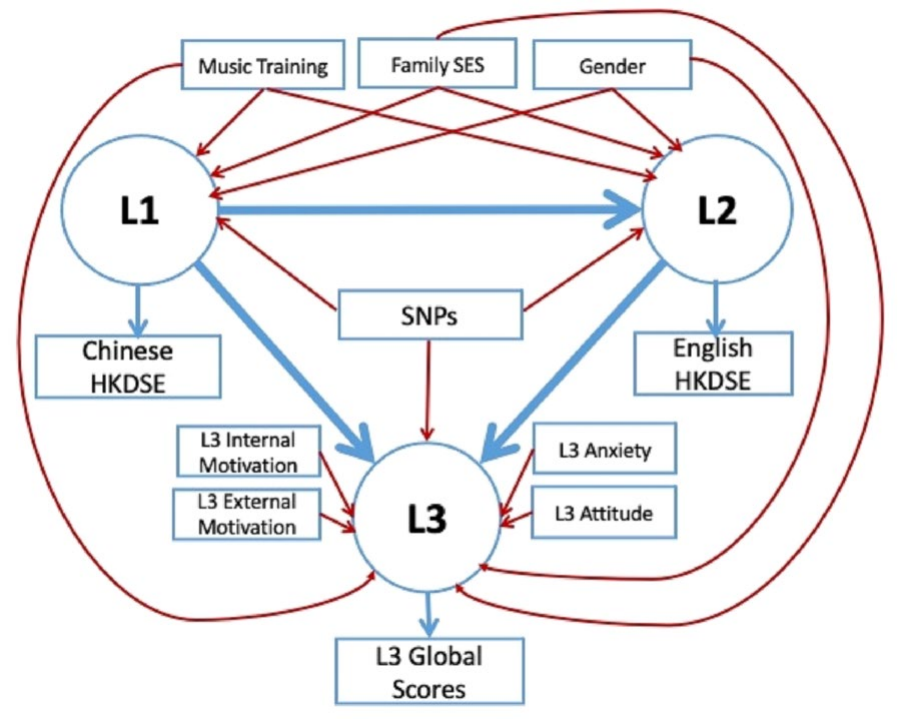
Metamodel of the structural equation model (SEM). Language proficiency of L1, L2, and L3 was added as latent variables with HKDSE Chinese and English scores and L3 Global scores as indicators, respectively. The significant SNPs in the final models of the stepwise regression and all non-genetic factors were entered into the SEM.

We conducted two types of analyses to evaluate our hypotheses (see “Methods and materials” for details). First, we used stepwise regression to evaluate genetic (all 28 SNPs) and non-genetic (e.g., gender) contributions to each language (L1, L2 or L3) in three models. This method allows us to determine unique variance explained by genetic and non-genetic factors for each language. However, a weakness of this approach is that we cannot simultaneously examine quantitatively whether the same genes or non-genetic factors also account for variance in the other two languages. Thus, followed by stepwise regression, we constructed a structural equation model (SEM) that included genetic variants that we found to contribute to any of the three languages we found in the regression models. These variants were entered into the SEM and their contribution to all three languages were tested simultaneously, along with non-genetic factors (Fig. 1). Because not all participants had all measures collected (genetic and non-genetic), we used listwise deletion to exclude those without complete data use in the regression analyses^57^ which resulted in fewer participants than the entire set (Tables 3, 4, 5, and 6 showed the number of participants included for each type of analysis).

**Table 3:** The final model of bi-directional stepwise regression analyses for L1 proficiency using the original dataset.

Predictor	Gene	Major allele	Estimate	Confidence intervals	Uncorrected *p*	FDR corrected *p*	Partial eta squared	∆R^2^
(Intercept)			7.49	4.51–10.47	**1.13e−06**			
**Gender**			**0.38**	**0.15–0.61**	0.001*^‡^	0.012*^‡^	**0.026**	**0.022**
**Family SES**			**− 0.01**	**− 0.01 to − 0.00**	0.008*^‡^	0.028*^‡^	**0.017**	**0.014**
rs6980093	*FOXP2*	*A*	0.12	− 0.03 to 0.26	0.110	0.121	0.006	0.003
**rs1800497**	***DRD2***	***G***	**0.21**	**0.06–0.35**	0.005*^‡^	0.025*^‡^	0.**020**	**0.016**
rs3765502	*DCDC2*	*T*	− 0.65	− 1.38 to 0.08	0.082	0.121	0.007	0.004
**rs6940827**	***DCDC2***	***G***	**− 0.87**	**− 1.62 to − 0.13**	0.022*^‡^	0.049*^‡^	**0.013**	**0.009**
rs2255526	*DIP2A*	*A*	0.15	− 0.03 to 0.34	0.106	0.121	0.006	0.004
rs6803202	*ROBO1*	*C*	− 0.11	− 0.25 to 0.04	0.162	0.162	0.005	0.002
rs9722	*S100B*	*G*	0.15	− 0.01 to 0.31	0.074	0.121	0.008	0.005
rs1087266	*DCDC2*	*A*	− 0.63	− 1.38 to 0.11	0.094	0.121	0.007	0.004
**rs6456593**	***DCDC2***	***C***	**− 0.20**	**− 0.36 to − 0.04**	0.012*^‡^	0.033*^‡^	0.**015**	**0.012**

Gender, family SES, and three SNPs (rs1800497, rs6940827, rs6456593) are independently associated with L1 HKDSE grades. The original model included gender (Female = 1; Male = 0), music training (Yes = 1; No = 0), family SES, and the 28 hypothesized SNPs. * indicates *p* < 0.05 (uncorrected); ^‡^represents significant associations after FDR corrections for multiple comparisons.

Observations: 421.

R^2^/R^2^ adjusted: 0.119/0.096, *p* = 2.215e−07.

Significant values are in bold.

**Table 4:** The final model of bi-directional stepwise regression analyses for L2 proficiency.

Predictor	Gene	Major allele	Estimate	Confidence intervals	Uncorrected *p*	FDR corrected p	Partial eta squared	∆R^2^
(Intercept)			4.22	3.60–4.83	** < 2e−16**			
Gender			0.21	0.01–0.40	0.040*	0.060	0.010	0.007
**Music**			**0.43**	**0.19–0.66**	0.0003*^‡^	0.002*^‡^	**0.031**	**0.026**
**Family SES**			**0.01**	**0.01–0.02**	1.27e−06 *^‡^	1.11e−05*^‡^	**0.056**	**0.050**
rs2538976	*CNTNAP2*	T	0.13	− 0.04 to 0.29	0.125	0.153	0.006	0.003
**rs2538991**	***CNTNAP2***	**C**	**0.20**	**0.02–0.38**	0.026*^‡^	0.046*^‡^	**0.012**	**0.008**
**rs6980093**	***FOXP2***	**A**	**0.27**	**0.05–0.49**	0.016*^‡^	0.036*^‡^	**0.014**	**0.010**
**rs1852469**	***FOXP2***	**A**	**− 0.34**	− **0.57** to **0.12**	0.003*^‡^	0.009*^‡^	**0.021**	**0.017**
rs4599626	*DCDC2*	C	− 0.12	− 0.28 to 0.05	0.155	0.155	0.005	0.002
rs9461045	*KIAA0319*	T	− 0.10	− 0.24 to 0.03	0.136	0.153	0.005	0.003

Music training, family SES, and three SNPs on *CNTNAP2* (rs2538991) and *FOXP2* (rs6980093, rs1852469) are independently associated with L2 HKDSE grades. The original model included gender (Female = 1; Male = 0), music (Yes = 1; No = 0), family SES, and the 28 hypothesized SNPs. * indicates *p* < 0.05 (uncorrected); ^‡^represents significant associations after FDR corrections for multiple comparisons.

Observations: 421.

R^2^/R^2^ adjusted: 0.138/0.119, *p* = 6.72e−10.

Significant values are in bold.

**Table 5:** The final model of bi-directional stepwise regression analyses for L3 proficiency with motivation variables included as additional predictors.

Predictor	Gene	Major allele	Estimate	Confidence intervals	Uncorrected p	FDR corrected p	Partial eta squared	∆R^2^
(Intercept)			− 0.01	− 0.35 to 0.32	0.939			
External			− 0.06	− 0.14 to 0.02	0.133	0.154	0.004	0.002
**Internal**			**0.22**	**0.14–0.30**	6.07e−08*^‡^	3.642e−07*^‡^	**0.057**	**0.054**
rs2538991	*CNTNAP2*	*C*	0.09	− 0.03 to 0.21	0.141	0.154	0.004	0.002
rs4680	*COMT*	*G*	− 0.09	− 0.22 to 0.04	0.154	0.154	0.004	0.002
rs6456593	*DCDC2*	*C*	− 0.14	− 0.26 to − 0.02	0.026*	0.052	0.010	0.007
**rs9722**	***S100B***	***G***	**0.15**	**0.03 to − 0.27**	0.015*^‡^	0.036*^‡^	**0.011**	**0.009**

Internal motivation *and S100B* (rs9722) are independently associated with L3 Global Scores. The original model included gender (Female = 1; Male = 0), music training (Yes = 1; No = 0), family SES, external motivation, internal motivation, attitude, and anxiety, and 28 SNPs. * indicates p < 0.05 (uncorrected); ^‡^represents significant associations after FDR corrections for multiple comparisons.

Observations: 510.

R^2^/R^2^ adjusted: 0.088/0.077, *p* = 2.614e−08.

Significant values are in bold.

**Table 6:** Path coefficients of structural equation models (SEMs) for L1, L2, and L3.

Language	Path	Major allele	Gene	Unstandardized	Standardized	z value	P value	95% CI
L1	**Gender**			**0.365**	**0.146**	**3.072**	**0.002**	**[0.132–0.599]**
	**Family SES**			**− 0.008**	**− 0.122**	**− 2.500**	**0.012**	**[− 0.015 to − 0.002]**
	Music			0.067	0.024	0.431	0.666	[− 0.238 to 0.372]
	rs9722	G	*S100B*	0.068	0.041	0.924	0.355	[− 0.076 to 0.213]
	rs2538991	C	*CNTNAP2*	0.024	0.015	0.290	0.772	[− 0.136 to 0.183]
	rs6980093	A	*FOXP2*	0.023	0.014	0.173	0.862	[− 0.234 to 0.28]
	rs1852469	A	*FOXP2*	0.085	0.052	0.627	0.531	[− 0.182 to 0.352]
	**rs1800497**	**G**	***DRD2***	**0.171**	**0.110**	**2.273**	**0.023**	**[0.023–0.318]**
	**rs6940827**	**G**	***DCDC2***	**− 0.252**	**− 0.123**	**− 2.659**	**0.008**	**[− 0.438 to − 0.066]**
	rs6456593	C	*DCDC2*	0.093	0.057	1.172	0.241	[− 0.063 to 0.249]
**L2**	**L1**			**0.224**	**0.253**	**5.411**	**6.258e−8**	**[0.143–0.306]**
	Gender			0.160	0.072	1.648	0.099	[− 0.030 to 0.350]
	**Family SES**			**0.015**	**0.256**	**5.477**	**4.326e−8**	**[0.010–0.021]**
	**Music**			**0.370**	**0.150**	**2.807**	**0.005**	**[0.112–0.629]**
	rs9722	G	*S100B*	− 0.064	− 0.043	− 1.052	0.293	[− 0.184 to 0.056]
	**rs2538991**	**C**	***CNTNAP2***	**0.133**	**0.094**	**1.996**	**0.046**	**[0.002–0.265]**
	rs6980093	A	*FOXP2*	0.157	0.113	1.548	0.122	[− 0.042 to 0.356]
	**rs1852469**	**A**	***FOXP2***	**− 0.246**	**− 0.169**	**− 2.224**	**0.026**	**[− 0.463 to − 0.029]**
	rs1800497	G	*DRD2*	− 0.047	− 0.034	− 0.784	0.433	[− 0.164 to 0.070]
	rs6940827	G	*DCDC2*	− 0.043	− 0.023	− 0.516	0.606	[− 0.205 to 0.120]
	rs6456593	C	*DCDC2*	− 0.085	− 0.059	− 1.396	0.163	[− 0.205 to 0.035]
L3	L1			0.008	0.009	0.164	0.870	[− 0.084 to 0.099]
	**L2**			**0.289**	**0.295**	**5.259**	**1.447e−7**	**[0.181–0.397]**
	Gender			− 0.103	− 0.048	− 1.19	0.234	[− 0.273 to 0.067]
	Family SES			− 0.005	− 0.085	− 1.907	0.056	[− 0.010 to 0.000]
	Music			− 0.108	− 0.045	− 1.14	0.254	[− 0.295 to 0.078]
	Attitude			− 0.018	− 0.02	− 0.498	0.619	[− 0.091 to 0.054]
	Anxiety			0.026	0.029	0.748	0.455	[− 0.043 to 0.095]
	External			− 0.033	− 0.034	− 0.858	0.391	[− 0.108 to 0.042]
	**Internal**			**0.229**	**0.243**	**5.755**	**8.653e−9**	**[0.151–0.307]**
	**rs9722**	**G**	***S100B***	**0.163**	**0.112**	**2.871**	**0.004**	**[0.052–0.274]**
	rs2538991	C	*CNTNAP2*	0.044	0.032	0.78	0.435	[− 0.066 to 0.154]
	rs6980093	A	*FOXP2*	− 0.185	− 0.135	− 1.908	0.056	[− 0.376 to 0.005]
	rs1852469	A	*FOXP2*	0.145	0.102	1.434	0.152	[− 0.053 to 0.343]
	rs1800497	G	*DRD2*	− 0.079	− 0.058	− 1.376	0.169	[− 0.191 to 0.033]
	rs6940827	G	*DCDC2*	0.062	0.035	0.913	0.361	[− 0.071 to 0.194]
	**rs6456593**	**C**	***DCDC2***	**0.150**	**0.106**	**2.412**	**0.016**	**[0.028–0.271]**

Gender and Music were coded as dummy variables with 1 = Female, 0 = Male; 1 = Have received at least 1 year of musical training, 0 = Have received less than 1 year of musical training or have not received any musical training at all. Both unstandardized and standardized beta coefficients between the two variables indicated by the path were reported. L1, L2, and L3 are latent variables of language proficiency with Chinese HKDSE grades, English HKDSE grades, and L3 Global scores as their indicators, respectively. In total, 609 participants were included in the SEM.

Significant values are in bold.

### Stepwise regression models

Stepwise procedure in both directions was implemented to determine which hypothesized SNPs (if any) significantly explained the variation in language proficiency. In the first step, all 28 hypothesized SNPs were included as predictors of language proficiency, along with non-genetic variables. The final model had the best combination of independent variables for predicting the language proficiency. Gender (*∆R*^2^ = 0.02, FDR corrected *p* = 0.012), family SES (*∆R*^2^ = 0.01, FDR corrected *P* = 0.028), two SNPs of *DCDC2* (rs6456593, rs6940827) (*∆R*^2^ = 0.01, FDR corrected *P* = 0.033; *∆R*^2^ = 0.01, FDR corrected *P* = 0.049), and one SNP of *DRD2* (rs1800497) (*∆R*^2^ = 0.02, FDR corrected *P* = 0.025) were significantly predicting L1 proficiency (Table 3). Music training (*∆R*^2^ = 0.03, FDR corrected *P* = 0.002), family SES (*∆R*^2^ = 0.05, FDR corrected *P* < 0.001), two SNPs of *FOXP2* (rs1852469, rs6980093) (*∆R*^2^ = 0.02, FDR corrected *P* = 0.009; *∆R*^2^ = 0.01, FDR corrected *P* = 0.036) and one SNP of *CATNAP2* (rs2538991) *(∆R*^2^ = 0.01, FDR corrected *P* = 0.046) were significant predictors of L2 proficiency (Table 4). Internal motivation (*∆R*^2^ = 0.05, FDR corrected *P* < 0.001) and *S100B* (rs9722) (*∆R*^2^ = 0.01, FDR corrected *P* = 0.046) were significant predictors of L3 proficiency (Table 5). Thus, for L1, the combined unique variances explained by common variants and non-genetic factors were 3.7% and 3.6%, respectively. For L2, they were 3.5% and 7.6%, respectively; and for L3, they were 0.9% and 5.4%, respectively.

### Structural equation modelling (SEM)

The stepwise regression approach reported above provided information about which ones of the 28 hypothesized genetic variants as well as non-genetic factors contributed to each language individually. In order to examine the contribution of genetic and non-genetic factors simultaneously for the three languages, we used SEM^58^ (see Fig. 1 for the metamodel). The SEM provided a statistically good fit, as indicated by the root mean square error of approximation (RMSEA) = 0.000 [*CI* 0.000–0.045], the standardized root mean square residual (*SRMR*) = 0.011, the robust Comparative Fit Index (*CFI*) = 1.000, the robust Tucker-Lewis Index (*TLI*) = 1.040, and the Yuan–Bentler scaling correction factor = 1.024. Table 6 presents path coefficients that represent the estimates of the connection strengthen between a unit change in genetic and non-genetic factors and the latent language proficiency variables. A positive coefficient means a unit increase in these factors leads to a direct and proportional increase in language proficiency, while a negative coefficient means that an increase in these factors leads to a direct and proportional decrease in language proficiency. We found that L1 proficiency was positively associated with Gender (standardized path coefficient 0.146) and *DRD2* (rs1800497) (standardized path coefficient 0.110), but negatively associated with Family SES (standardized path coefficient − 0.122) and *DCDC2* (rs6940827) (standardized path coefficient − 0.123). L2 proficiency was positively associated with L1 proficiency (standardized path coefficient 0.253), Family SES (standardized path coefficient 0.256), music (standardized path coefficient 0.150), *CNTNAP2* (rs2538991) (standardized path coefficient 0.094), but negatively associate with *FOXP2* (rs1852469) (standardized path coefficient − 0.169). L3 proficiency was positively associated with L2 proficiency (standardized path coefficient 0.295), internal motivation (standardized path coefficient 0.243), *S100B* (rs9722) (standardized path coefficient 0.112), and *DCDC2* (rs6456593) (standardized path coefficient 0.106). Generally speaking, the SEM results converged with the stepwise regression results, even when proficiency levels for all three languages were considered together.

## Discussion

We found little overlap in the genetic associations among the three languages that our participants learned at different times in life. This pattern of results can be seen when the three languages were examined individually or simultaneously. Instead, we found that different common genetic variants contribute to explaining variance of the three languages. The effects of genes on language seem to be language specific and are stronger for native than foreign languages. By contrast, the effects of non-genetic factors seem to be stronger for foreign than native languages.

We found two genes that contributed to explaining variance in L1 ability in our stepwise regression, *DCDC2* and *DRD2*. Importantly, the significant *DCDC2* variants were those found in other studies of Chinese, including rs6456593^34^, and rs6940827^35^, each contributing to about 1% of the variance in L1. *DRD2* (rs1800497) was found to contribute significantly to about 1.6% of variance in our study. In a previous study, the same variant was found to explain variance in bilingual proficiency^52^, which confirmed the results of a previous artificial language learning study where young adults learned a morpho-phonological grammar^42^. We found two different genes associated with L2, namely *CNTNAP2* and *FOXP2*, which combined explained about 3.5% of variance*. CNTNAP2* (rs2538991), which is downregulated by *FOXP2*, is associated with non-word repetition in English^5^. Non-word repetition is a predictor of language impairment in English-speaking children^59^. Interestingly, in Chinese, non-word repetition did not predict language impairment^60^. Thus, the association of *CNTNAP2* (rs2538991) with English only may support the language-specific hypothesis. The specific genetic variants of *FOXP2* that we found to be associated with L2 included rs6980093, which was associated with verbal fluency (naming as many words as possible in a semantic category within 60 s) in two Italian samples^45^, and rs1852469, which has been associated with speech sound disorders in a Chinese population^44^. Compared to L1, the genetic effects on L3 is much weaker. For the common variants examined, *S100B* (rs9722) was the only significant contributor to L3 proficiency in the stepwise regression analysis, which explained about 1% of variance. *S100B* are highly expressed in the hippocampus^61^. Its association with the learning of a new language is consistent with the role of declarative memory in early stages of language learning^62^. The pattern of results of the SEM converged with those of the stepwise regression, except that rs6456593 was also found to be associated with L3 but not L1. This difference does not change the preliminary conclusions of the study.

Table 7 summarizes the SNPs that we found to be significantly associated with language phenotypes in the present study. The risk alleles we found in the present study and other relevant studies are also listed. For the most part, our findings are consistent with those reported in the literature with two exceptions. For rs1852469 and rs2538991, the allele which we found to be associated with weaker language ability was opposite of what was found in Zhao et al.^44^ and Vernes et al.^5^, respectively. In both cases, the allele frequencies in our sample were different from what was reported in those studies. While the allele frequencies we found for rs1852469 was consistent with what was reported in dBSNP (https://www.ncbi.nlm.nih.gov/snp/) (A>T), the opposite was found in Zhao et al.^44^ (T>A), even though both samples were East Asian. For rs2538991, the allele frequencies were roughly equal for the European population that Vernes et al.^5^ studied, but for our sample of East Asian, the A allele was clearly the minor allele.

**Table 7:** Risk alleles of SNPs that were reported to be linked with language abilities in the present and in the literature.

SNPs	Gene	Risk allele in our study	Risk allele in the literature	Phenotypes	Population	Sample size	References
rs1800497	*DRD2*	A	A	Grammatical rule learning	European (USA)	22 adults	^42^
rs1852469	*FOXP2*	A	T	Speech sound disorder	East Asian (Han Chinese)	150 patients with speech sound disorder and 140 healthy controls	^44^
rs6980093	*FOXP2*	G	G	Expressive language , fluency	European (Italian)	699 population-based cohort and 572 children with developmental dyslexia	^45^
rs2538991	*CNTNAP2*	A	C	Specific Language Impairment (SLI)	European (USA)	847 members of 184 families	^5^
rs6456593	*DCDC2*	C	C	Developmental dyslexia (DD)	Asian (Uyghur)	392 Uyghur children aged 8–12 years old	^34^
rs6940827	*DCDC2*	G	G	Developmental dyslexia (DD)	Asian (Han Chinese)	54 trios aged between 5 and 16 year	^35^
rs9722	*S100B*	A	A	Developmental dyslexia (DD)	European (Finland, Germany and Sweden)	100 participants with DD	^51^

The amount of variance explained by any single SNP was about 1 to 2% in this study, which is seemingly large when compared to those effects found in GWAS studies (e.g., Okbay et al.^63^). Only 28 SNPs were examined in the present study, and it is likely that overlapping variance with other SNPs that we did not investigate would be revealed should a GWAS study was conducted. Furthermore, because our candidate SNPs have been studied extensively in other studies, they represent those of larger effects and our replication here speaks to that. In addition to these explanations, it is important to acknowledge that smaller studies such as this one often results in overestimation of effect sizes^64^ and even false positives.

Taken as a whole, the results may support the hypothesis that genetic associations are strongest for a specific language. Furthermore, genetic effects seem to be strongest for native than foreign languages. For L1, the amount of variance explained by genetic factors combined (3.7%) was much stronger than that of any one of the significant non-genetic factors, including gender^65^ (2.2% of variance explained) and family SES^16^ (1.4%). For L2, the best predictor was family SES^66^ (5%), followed by music training^18–20^ (2.6%). For L3, the best predictor was clearly the non-genetic factor of motivation (5.4%). Again, this finding is consistent with the results of previous non-genetic studies^55, 67–69^, which found motivation to be the best predictor of learning a new language.

It is worth noting that the effect of family SES on L1 is in the negative direction in our sample. This is likely a unique finding to learning L1 and L2 in Hong Kong. In a longitudinal study in school children in Hong Kong, family income only predicted L2 (English) but not L1 (Chinese) proficiency^70^. In early adulthood, this trend may lead to a negative association between family SES and L1 because of an emphasis on learning English for families of higher SES background, as learners from higher SES families are more likely to attend English-medium schools.

An important feature of our study is that we examined the genetic associations of three languages all within a single (Han Chinese) population and investigated the contributions of a group of genes that have found to be related to language. This design allows us to more clearly study how the same group of genes are associated with different languages and languages learned at different times, without contamination by the co-varying factors of population and language. As far as we know, Waye et al.^24^ are the only other researchers who have examined L1 and L2 within the same population. However, only the genetic variant rs3743205 of *DYX1C1* was studied. Vaughn and Hernandez^52^ also examined two languages but did not report association results for each language independently, focusing instead only on bilingual proficiency, a measure of the balance of two languages.

Our study contributes to the decades-long debate in language learning about whether native and foreign languages are learned primarily with the same mental mechanisms. Our two hypotheses were aligned with the Linguistic Coding Differences Hypothesis (LCDH)^71^ and the Fundamental Difference Hypothesis (FDH)^23, 72^. Under LCDH, a set of identical “core languages functions” such as phonological and syntactic processes are required for the successful learning of any languages at any time in life. In terms of genetics, this implies the same set of genetic variants for native and foreign languages. FDH hypothesizes an innate language learning system that is only accessible at the earliest time in life for learning an infant’s native language. Foreign language learning lacks access to this innate system. In genetic terms, it implies a group of genetic variants that are only associated with L1.

Wong et al.^73^ hypothesized that dopamine-related genes are linked to individual differences in language learning. Vaughn and Hernandez^52^ tested this hypothesis and found a significant association between the dopamine-related genes *COMT* (rs4680) and *DRD2* (rs1800497), and individual differences in achieving balanced bilingual proficiency. Wong et al.^42^ who used an artificial language in laboratory conditions rather than an authentic language, found a significant association between *DRD2* (rs1800497) and the learning of morphophonology. Stein et al.^74^ found a significant association between several SNPs of *DRD2* (including rs1800497) and measures of native language but only the vocabulary measure reached statistical significance after correction for multiple comparisons. Nevertheless, the findings from these previous studies are consistent with those of the present study. The dopamine hypothesis concerns a language universal mechanism. Future research will need to explore why the present study only found a significant association with native language.

Our study has several limitations. First, although the genetic variants we examined were those that have been reported (and sometimes replicated) in research studies during the past two decades and are the most promising candidates for language, many more potential genetic variants remain to be examined. It is very likely that those genetic variants may show an overlap across three languages. But based on the best available information we have about genes and language, we designed our study and found interpretable findings to confirm one of the two hypotheses. A GWAS with a very large sample size is needed in the future. Second, although we have found differences in genetic associations across languages, it is still unclear whether they occur because of language features or because they are languages learned at different points in life. Our evidence provides support for both explanations. A much larger-scale study with a much larger sample size in the future would control for the different grouping of languages and when they are learned, which would allow for a more precise delineation of these two factors. Third, only Han Chinese participants were studied. Future research will need to sample different populations (see Carrion-Castillo et al.^75^ and Becker et al.^76^ for examples of studies of European samples) who may have different, subtle genetic differences which may not occur in such a restricted sample. Fourth, we did not collect data on participants’ time on L3, which may explain some of the variance in L3 proficiency.

In a unique sample of Han Chinese participants who have learned three different languages, we found differences in genetic associations that depend on the specific language and when the language is learned. Individual differences in L1 seem to be more highly associated with language-related genes, especially those that have been found to be related to impairment of Chinese. L2 seems to be more closely related to both genetic and non-genetic factors (musical background and family SES). L3 is most strongly related to the motivation of the learners who learn the new language. Our results did not lend support to the hypothesis that a common set of genetic factors contribute to all language learning. It is likely that language learning at different times in life requires different processing demands^77^, which are underlined by different neurogenetic factors. It is also likely that different language features require different processing demands and, as a result, different neurogenetic factors contribute to different languages^54^. The present study should be viewed as a preliminary step towards exploring the two primary hypotheses. Future research of a much larger scale is required to further explore the nature of genes and language.

## Methods and materials

### Participants

We recruited a total of 940 participants (696 females) between 18 to 25 years of age (*Mean* = 19.98, *SD* = 1.28) for our study through mass emails and advertisements in their language classes, after obtaining permission from the class teachers. Written informed consent was obtained from all participants. The research protocol was approved by the Joint Chinese University of Hong Kong—New Territories East Cluster Clinical Research Ethics Committee and the research was performed in accordance with the Declaration of Helsinki. All participants were native speakers of Cantonese of Han Chinese descent without any self-reported neurological or psychiatric disorders. They all scored within normal limits (at least 85) of the nonverbal intelligence measured by the Test of Nonverbal Intelligence (4th Ed)^78^ and passed the hearing screening at the frequencies of 500, 1 k, 2 k and 4 k Hz at 30 dBH. All learned English as L2, and French, German, or Spanish as L3. Because these participants enrolled in this study over a 4-year period, not all variables were collected from every participant. Some data was also missed due to fatigue, coding errors and genotyping failures. Table 2 presents descriptive measures for the different participant variables.

### Questionnaires

We collected demographic information on the participants, including their gender, date of birth, language background, family socioeconomic status (SES), and musical experiences. Family SES was determined by following the Hollingshead index^79^ by coding parents’ educational levels and occupational prestige. Participants also completed the Modern Language (ML) Learner Questionnaire^80^ to indicate their internal motivation, external motivation, anxiety, and attitudes to learning the L3. A data reduction process was used to derive four metrics related to this questionnaire (see SI).

### Proficiency of L1, L2, and L3

The L1 and L2 proficiency of participants were measured by the composite scores of each of the Chinese and English language subjects of the Hong Kong Diploma of Secondary Education Examination (HKDSE), the public examination for university entrance in Hong Kong, administered by the Hong Kong Examinations and Assessment Authority (HKEAA). HKDSE implements an annual calibration exercise to ensure that scores across years reflect the same levels of performance^81^. For both Chinese and English, the composite scores were calculated using subtests on reading, writing, speaking, and listening skills on a scale from 1 (lowest) to 7 (highest).

To obtain an overall measure of L3 proficiency, we collected laboratory-based and classroom-based data which covered reading, writing, speaking, and listening abilities for each third language, similar to L1 and L2. Laboratory-based measures included three types of data. First, a sample of passages read aloud from the “*Frog, Where Are You?”* story^82^ was transcribed, morphosynatically tagged, and analyzed using the CLAN program of the TalkBank project^83^. Second, the pronunciation of speech production was assessed by native speakers based on excerpts from the storytelling sample. Third, lexical access was calculated by using the accuracy rates of a picture naming task. Classroom-based measures were participants’ z-transformed exam scores of the L3 class. The final L3 proficiency index, known as the L3 Global score, was calculated by using the Principal Component Analysis based on these measures. Details regarding to data collection, analysis, and reduction procedures for L3 proficiency are given in SI Materials and Methods.

### Genes and SNP genotyping

Saliva samples were collected using Oragene (DNA Genotek) and used to extract the genomic DNA of participants. A NanoDrop Spectrophotometer was used to quantify Extracted DNA samples, and was normalized to 5 ng/μl for use in genotyping. A commercially available Sequenom MassARRAY platform was used to genotype the SNPs. Table 1 presents the allele frequencies of our sample. For the most SNPs, the allele frequencies in our sample are consistent with those reported by the dbSNP database published by the National Center for Biotechnology Information (US) (https://www.ncbi.nlm.nih.gov/snp/) for East Asians.

In selecting our genetic candidates, our focus was on individual differences of language functions on a continuum and their association with common genetic variants, rather than rare forms of neurodevelopmental disorders or disorders that lead to language impairment as a secondary condition. SNPs of *FOXP2* were included so far as they were common variants and were associated with speech^44^. We conducted a literature search for studies that had investigated individual differences in typical language functions or language impairment. For genetic variants associated with language impairment, we only considered language impairment as a primary condition (Developmental Language Disorder), excluding studies of autism, intellectual disability, and other neurodevelopmental disorders where language impairment of any modality is a secondary condition^84–90^. We also excluded studies that examined rare deletions^7^, along with studies of genetic variants that are linked to stuttering without other traits related to abstract linguistic structures^91^. We only included variants of *CNTNAP2* that have been associated with primary language conditions^5^. *CNTNAP2* has been associated with language functions in Autism Spectrum Disorder (ASD) in children of European backgrounds^92^. In Chinese children with ASD, there are conflicting findings regarding the role of *CNTNAP2* polymorphisms^93, 94^. Given these uncertainties, SNPs that were associated with language in ASD but not language as a primary condition were excluded. We also excluded SNPs due to linkage disequilibrium with other SNPs in the study. Linkage disequilibrium (LD) among the SNPs on the same chromosome was calculated using snpStats^95^ package of R^96^ (see Fig. S5 for the LD results). In the end, based on the results of previous studies which reported associations with language functions, we composited a list of 28 SNPs as our candidates (see Table 1 for the references).

### Statistical analysis

Because each analytic method has its own strengths and limitations, we opted to use multiple methods for our data analysis. Based on the practice of previous studies, we chose two methods: stepwise regression and structural equal modeling (SEM). We began our analysis with stepwise regression. For each language of a stepwise regression model, we used the 28 SNPs as predictors, and used family SES, gender, and musical training as non-genetic predictors. For L3, we also analyzed the data with motivation measures as additional predictors. Standard linear additive SNP encoding was used to code the alleles. The major alleles were given a value of 2, the heterozygous alleles a value of 1, and the minor alleles a value of 0. Thus, a positive statistical relationship between SNP and language means a higher load of the major alleles for better language.

#### Stepwise regression

We included all 28 SNPs and non-genetic variables (gender, music training, and family SES for L1 and L2; these factors and motivational factors for L3) in stepwise regression models for L1, L2, and L3 separately. Stepwise regression is a method of fitting regression models in which the choice of predictive variables is made by an automatic procedure. The final model had the best combination of independent variables for predicting the dependent variables. For all models, stepwise procedure in both directions was implemented via MASS package^97^ of R^96^ to remove and add predictors based on their improvement to the Akaike information criterion (AIC). Final models of stepwise regression included all predictors that showed improvement to the AIC. Statistical significance of each variable was also indicated by the false discovery rate (FDR) corrected *p* values, which were calculated using the Benjamini–Hochberg method.

#### Structural equation modelling

To quantify the statistical relationships of language proficiency and hypothesized SNPs, we fitted a structural equation model (SEM) using the lavaan package^98^ of R^96^. Demographic characteristics, including gender, music training, and family SES, and genetic variants that were associated with each language separately from stepwise regression models were considered independent variables in the data analysis. Proficiency in each language was treated as a latent variable. In the metamodels, we hypothesized that both non-genetic (e.g., gender, music training, and family SES) and genetic variables had effects on proficiency of each language (Fig. 1). For L3, motivation was additionally associated with proficiency^56^. As proficiency levels among languages might be related as found in our recent study^55^, those relationships were also accounted in the SEM. We used the full information maximum likelihood (FIML) to account for missing data and robust SEs accounting for non-normality. The goodness of fit for the tested model was established by the following indices: (i) χ^2^ test with an estimated significance level *P* ≥ 0.05, (ii) *χ*^2^*/df* < 2, (iii) robust root mean square error of approximation (robust *RMSEA*) < 0.05 and an upper limit of the 95% confidence interval (*CI*) for robust *RMSEA* < 0.08, (iii) robust comparative fit index (robust *CFI*) and robust Tucker–*Lewis* Index (robust *TLI*) with values ≥ 0.90, and (iv) standardized root mean square residual (SRMR) with a value lower than 0.10. We reported both unstandardized and standardized path coefficients (Table 6).

## Supplementary Information

Supplementary Information.

## Data Availability

All data needed to evaluate the conclusions in the paper are present in the paper and/or Supplementary Information. The numeric data and analysis scripts of this study will be available at *Open Science Framework* (https://osf.io/vkgmd/).
