# Supplementary material for: Contributions of common genetic variants to specific languages and to when a language is learned
Source: Sci Rep. 2022 Jan 12;12:580. doi: 10.1038/s41598-021-04163-1 (PMC8755716; doi:10.1038/s41598-021-04163-1)
Supplement: Supplementary file 1 — Supplementary Information. [file 41598_2021_4163_MOESM1_ESM.pdf]

Supplementary Information

SI Methods and Results

Motivation Questionnaire. The Modern Language (ML) Learner Questionnaire, adapted from Dörnyei and Ushioda (2009)[55], was used to measure learners' motivational and affective factors of third language learning. The original questionnaire measured 14 factors of language learning, including attitudes to L3 Community, cultural interest, and anxiety. In the first part of the questionnaire, participants were asked to indicate how far they agreed or disagreed with 49 statements (such as "I have to learn ML because I don't want to fail the ML course") on a 6-point Likert scale ranging from "Strongly disagree" to "Strongly agree". In the second part, participants were asked to answer 18 questions (such as "Do you always look forward to ML classes?") on a six-point Likert scale ranging from "Not at all" to "Very much". In Part One, we verified the Kaiser-Meyer-Olkin (KMO) measure that the overall Measure of Sampling Adequacy (MSA) = 0.87 and all variables having MSA above .50 cutoff point. We also checked the Bartlett's test of sphericity, $\chi 2 = 603$, $p < 0.001$. To reduce the dimensions, we conducted principal component analysis (PCA) with Varimax rotation for the two sections of the questionnaire separately. Two components that explained 36% of the variance were retained from Part One. One included factor related to extrinsic variables such as Ought-to L2 self and Family influence, and was labeled "external motivation", while the other covered items on Ideal L2 Self, and was therefore labeled "internal motivation" (Fig S1a). Similarly, questions in Part 2 also met the criteria for PCA, with all variables having MSA above .50 cutoff point and Bartlett's test of sphericity, $\chi 2 = 1936$, $p < 0.001$. Thus, two components which explained 43 % of the variance, namely "anxiety" and "attitude", were retained (Fig S1b).

L3 proficiency measures. As participants learned different third languages (French, German, and Spanish) and were enrolled in different classes (ranging from beginners to advanced learners), we collected both laboratory-based and classroom-based measures for the calculation of the L3 Global score as a comprehensive and valid measure of L3 proficiency across participants. We outline below the detailed methods of data collection, analysis, and reduction procedure.

L3 narrative measures. A children's wordless story book, "*Frog, Where Are You*?"[81] (Mayer, 1967) was used to elicit speech samples of storytelling. Participants were tested individually in a quiet booth. They were given a hardcopy of this book and were instructed to tell the story at their own pace, while a microphone recorded their speech. Native speakers transcribed the recordings and coded errors at the word-level and utterance-level. As a further reliability check, a second native speaker transcribed and coded 10% of the speech samples, and also double checked all transcripts. The transcripts were then checked again by research assistants. When there were discrepancies of transcription or coding, the first transcriber was contacted and resolved them with the second transcriber. The transcripts were then morphosyntactic ally tagged using the CLAN program of TalkBank[82]. Then, 15 indices of L3 narrative measures were extracted: (1-2) total number of utterances (all utterances; utterances after excluding unintelligible ones), (3-4) mean length of utterances (by words and by morphemes), (5) types of words, (6) tokens of words, (7) type and token ratio, (8)the number of words per minute, (9) density, (10) the number of verbs per utterances, (11) errors at word level, (12) errors at utterance level, (13) total number of words, (14) percentage of retraced words, and (15) percentage of repeated words.. Z-transformation was conducted for all indices at the class

level of each L3 language. In this way, we were able to keep the individual differences in L3 proficiency among participants, while removing the variance of class levels and L3 languages. To further reduce the dimensions of narrative measures, we conducted a principal component analysis (PCA) with varimax rotation. The Kaiser-Meyer-Olkin (KMO) measure verified the sampling adequacy for the analysis with an overall MSA = 0.79 and with all variables having MSA above .50 cutoff point. Bartlett's test of sphericity, $\chi 2 = 1{,}414$, $p < .001$, indicated that correlations between items were sufficiently large for PCA. In this analysis, we extracted two components that explained 54% of the variances (Figure S2)

L3 pronunciation ratings. Excerpts were taken from the beginning and the end of each storytelling recording for the rating of L3 pronunciation. Each excerpt was about 20-30 seconds long, and contained full sentences (after the exclusion of any initial pauses or false starts). Two counterbalanced lists were created for each L3 language, which were further divided into blocks of 30-40 excerpts per list. The excerpts were presented to native speakers of the L3 languages via Qualtrics.com. For each block, 16 native speakers were asked to indicate on a 9-point Likert scale the degree of native-like qualities of pronunciation. We averaged the ratings of the two excerpts at the beginning and each audio recording as the L3 pronunciation ratings of participants.

L3 language access. Language access of participants was examined using a body parts naming task using the Hawaii Assessment of Language Access battery (O'Grady, et al., 2009). 31 photographs of boy parts (e.g., hand, foot, ankle) were presented in a randomized order on a computer screen using the E-prime program. Participants were instructed to name each picture as

quickly as possible. When they finished naming the picture, they pressed a button on the response box to present the next stimulus. Native speakers of the target languages transcribed and judged whether correct names were provided for the pictures. We calculated the accuracy rates as the measure of L3 language access.

<u>*Classroom-based measures.*</u> Raw exam scores at the end of each academic term were obtained from the Department of Linguistics and Modern Languages for each participant. The mean and standard deviation of the exam for the entire class were also obtained. We then converted the raw exam score into a z score.

<u>*L3 Global scores.*</u> The final L3 Global score for each participant was calculated using the above measures, including two PCA components of the narrative production, the pronunciation ratings, the accuracy rates of picture naming, and the z-scores of the classroom exam. All measures were standardized across participants at the same language level of each L3. We then used the PCA with a varimax rotation to further reduce the dimensions by retaining one component. The Kaiser-Meyer-Olkin (KMO) measure verified the sampling adequacy for the analysis with an overall MSA= 0.60, with all variables having an MSA above .50 cutoff point, and Bartlett's test of sphericity, $\chi 2 = 20.73$, $p = 0.023$. An initial analysis was run to obtain eigenvalues for each component in the data. We took the loadings of the first component that had an eigenvalue of 1.56 and explained 31% of the variance as the L3 Global scores to mark the overall L3 proficiency of the participants (Figure S3). Figure S4 presents the Spearman correlations between the L3 Global scores and each measure of L3 proficiency.

Supplementary Figures and Tables

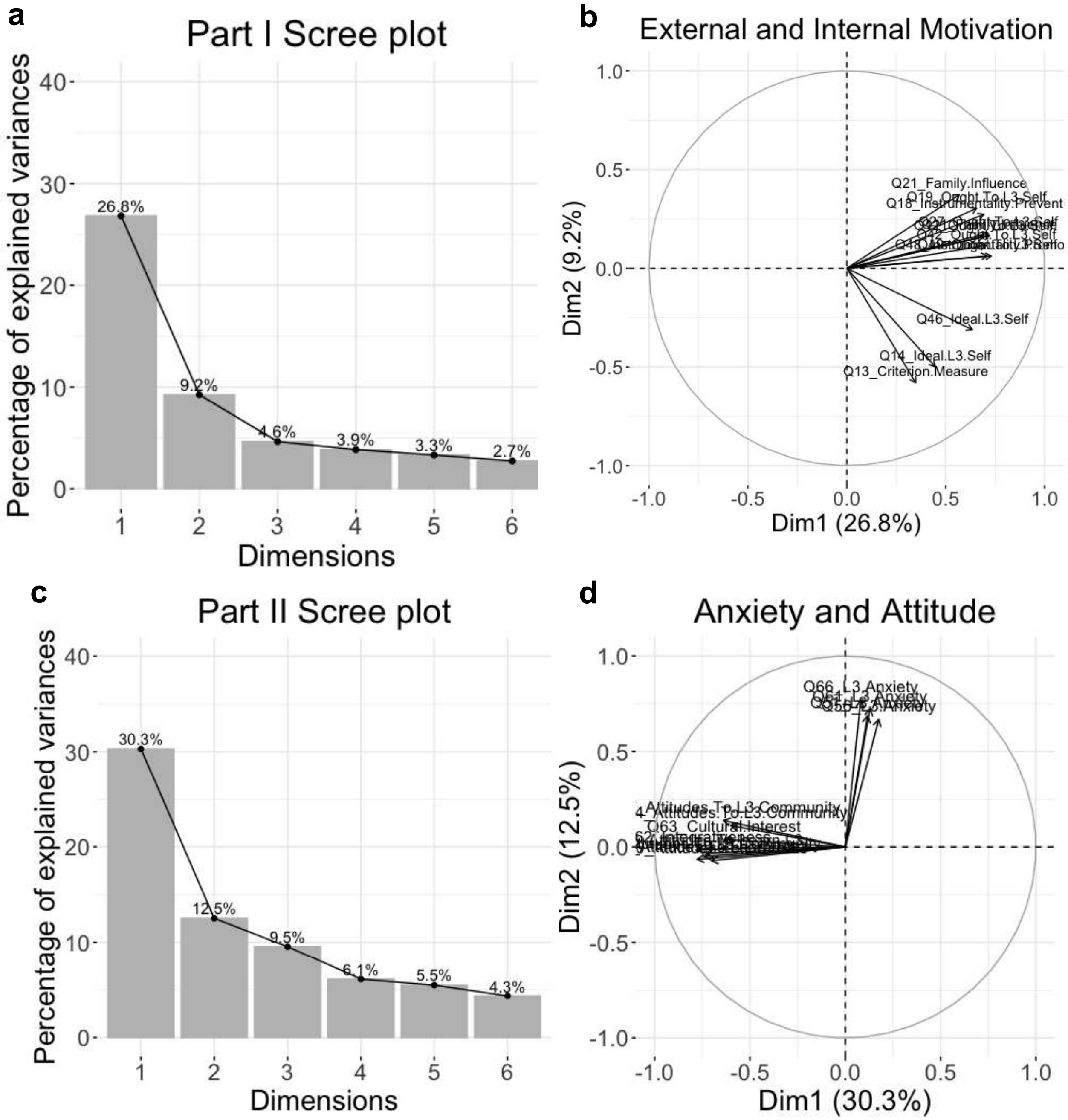

Fig.S1. S Principal component analysis (PCA) for the L3 learners' motivation questionnaire. a) Two components explained 36% of the total variances in Part I. b) Dimension 1 of Part I consisted of questions related to "ideal L3 self", labeled "internal motivation"; Dimension 2 of Part I consisted of questions related to "ought to L3 self", labeled "external motivation". c) two components explained 43% of the total variances. d) Dimension 1 of Part II was indicated by questions related to "anxiety", while Dimension 2 of Part II had questions related to attitudes toward the L3 community and L3 learning, labeled "attitude".

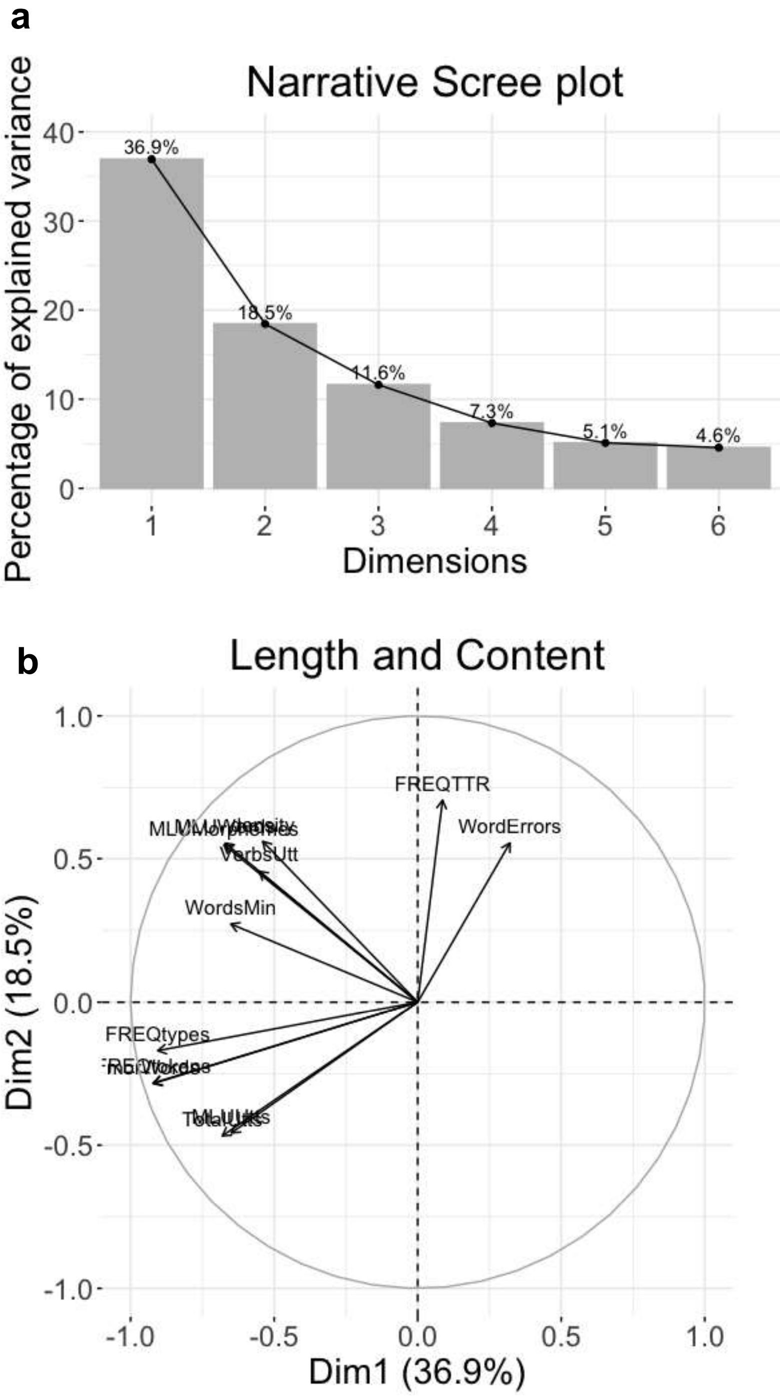

Fig.S2. Principal component analysis (PCA) for the L3 narrative measure. a) Scree plot. b) Two components explained 54% of the total variances. Dimension 1 was consistent of items related to mean length of utterances, and verbs per utterances, thus named as "content", while Dimension 2 was consisted of items related to total number of utterances and words, thus named as "length".

a

b

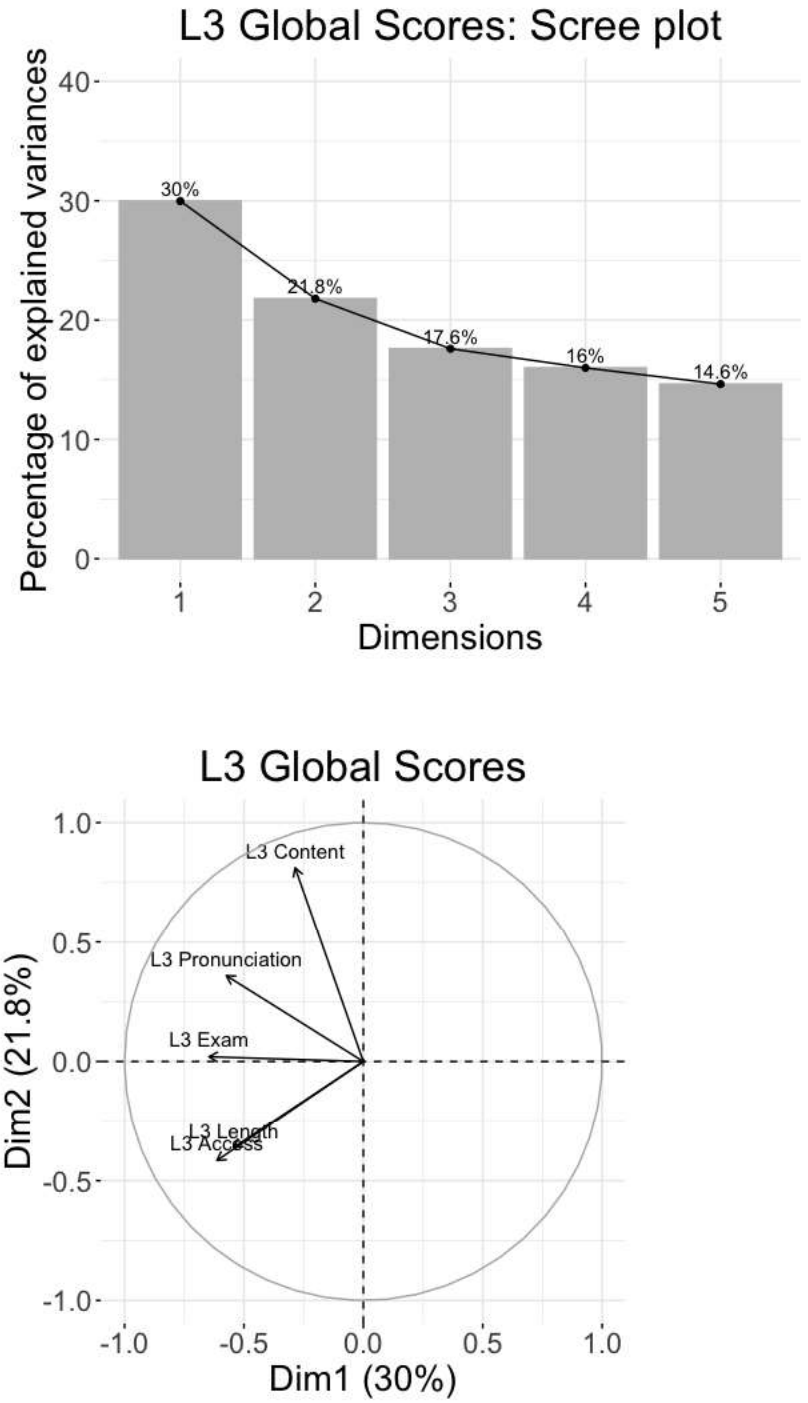

Fig.S3. Principal component analysis (PCA) for the L3 Global Scores. a) Scree plot b) The loadings of the first dimension explained 31% of the total variances, and were used as the L3 Global Scores.

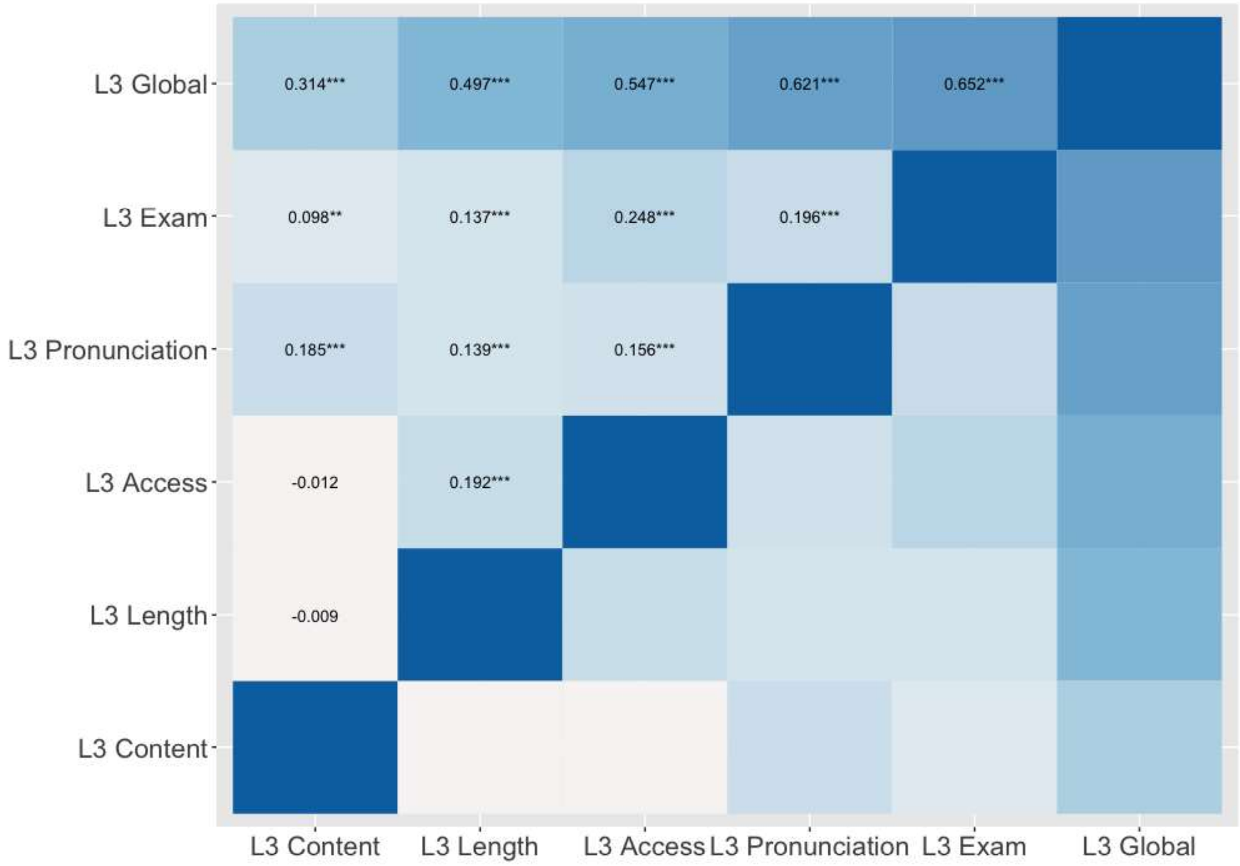

Fig.S4. The correlation matrix of L3 outcome measures. The values in the boxes indicate Spearman's rho correlation coefficients. The scatterplots in the boxes on the left side of the panel illustrate the bivariate correlations. * indicates $p < 0.05$ (uncorrected), ** $p < 0.01$, *** $p < 0.001$.

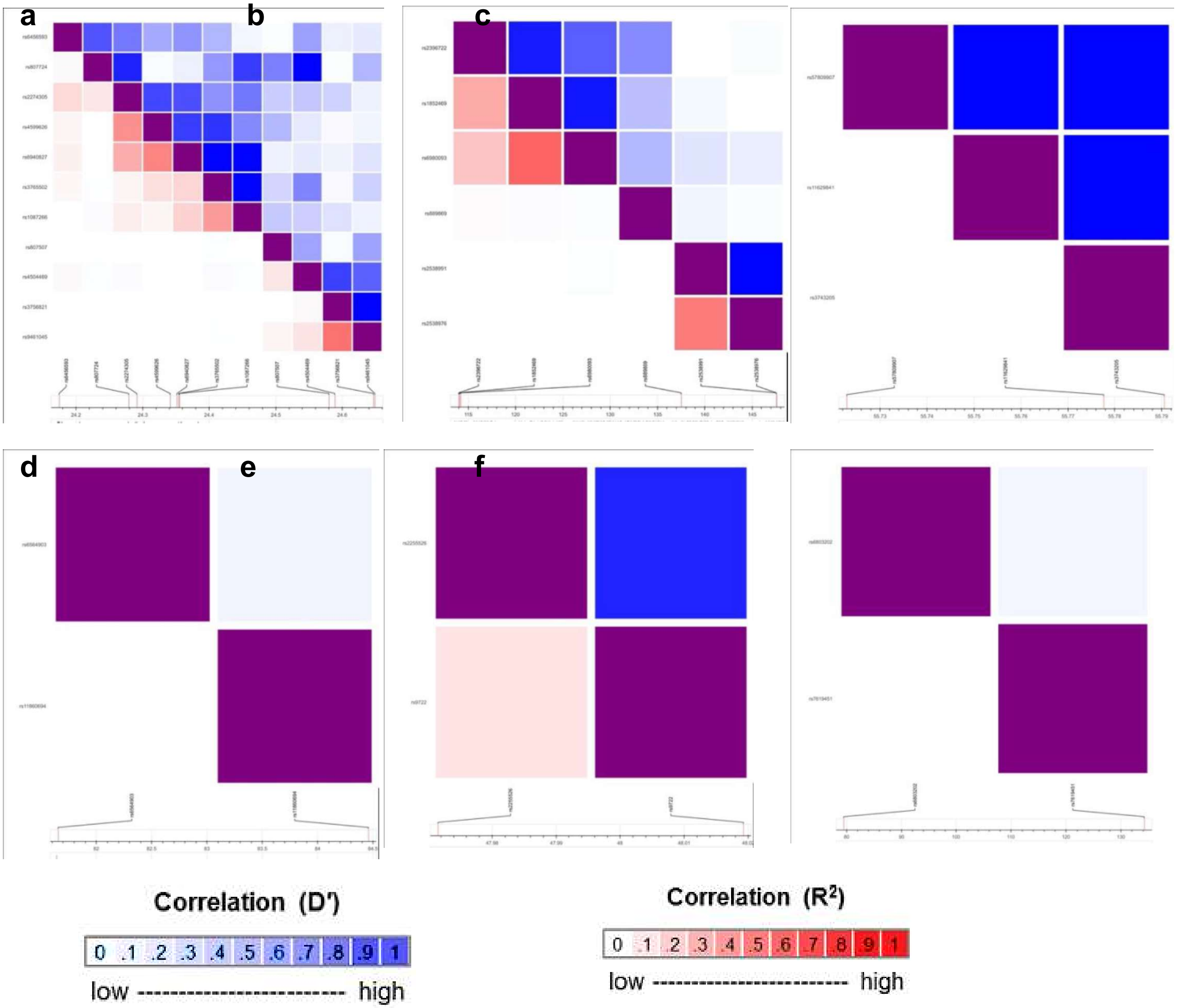

Fig.S5. Linkage disequilibrium (LD) among SNPs on the same chromosomes. R^2 and D' values were color-coded a) Chromosome 6 (*DCDC2* and *KIAA0319*). b) Chromosome 7 (*CNTNAP2*, *FOXP2*, and *DGKI*). c) Chromosome 15 (*DYX1C1*). d) Chromosome 16 (*CMIP, ATP2C2*). e) Chromosome 21 (*DIP2A, S100B*). f) Chromosome 3 (*CEP63, ROBO1*).
